# Effect of family empowerment health education on health behavior of young stroke patients based on a cross-theoretical model

**DOI:** 10.4314/ahs.v25i3.16

**Published:** 2025-09

**Authors:** Di Liu, Huimin Liang, Chunmei Wang

**Affiliations:** School of Nursing, Tianjin Medical University, Qixiangtai Road, Heping District, Tianjin 300070, China

**Keywords:** Cross-theoretical model, Family empowerment, Youth stroke, Behavioral stage changes, Caregiver feelings

## Abstract

**Purpose:**

This paper aimed to explore the effects of cross-theoretical model-based family empowerment health education on health behavior and blood pressure control in young stroke patients.

**Method:**

A total of 142 young stroke patients who met the inclusion and exclusion criteria, were admitted to the neurosurgery department of a Tertiary Grade A hospital in Tianjin. In detail, there were 71 cases each in the control group and experimental group. The control group was given routine health education intervention, while the experimental group was received family empowerment health education intervention according to the proposed cross-theoretical model. These two groups' health behaviors, behavioral stage changes, and blood pressure of the patients in the above-mentioned two groups before and after the intervention were compared systemically.

**Results:**

The score of healthy behavior in the experimental group was higher than that in the control group (P < 0.05), the behavioral stage change in the experimental group was better than that in the control group (P < 0.05), and the positive feeling of the primary caregivers of young stroke patients in the experimental group was much better than that in the control group (P < 0.01).

**Conclusion:**

Cross-theoretical model-based family empowerment health education could effectively improve patient health behavior and promote patients' behavioral change, and increase the positive perception of their primary caregivers.

## Introduction

The high incidence, disability and fatality rate of stroke in the world have brought great impact on medical services in various countries. However, with the continuous development of society and the increasing pressure of work and life, the incidence of stroke is getting younger year by year^1^. In the prevention and rehabilitation of stroke, health education is particularly important and has become an important task in the current prevention and treatment of stroke^2^. Previous studies^3^-^5^ have found that most of the risk factors for stroke onset and recurrence are associated with unhealthy behaviors. With the continuous development of comprehensive prevention and treatment of stroke, the health education model based on the cross-theoretical model has been understood by more and more people. The cross-theoretical model is a purposeful behavior change model. The cross-theoretical model is a behavioral stage change theory based on a variety of psychological theories^6^. So far, it has been widely used in the systematic research field of people's health behavior^7^-^8^. In particular, this model promotes the transition of individual behavioral stage changes by investigating the health education strategies corresponding to individuals at specific behavioral stages^8^. According to the patients' willingness and time to change, the whole process of behavior change is divided into five stages, and different behavior transformation strategies are adopted for individuals at different stages to promote the occurrence of behavior change^9^.

In light of these, this paper explored the effect of cross-theoretical model health education on the health behavior and blood pressure control of young stroke patients according to the specific requirements of young stroke patients at their different stages.

## Methods

### Study population

A total of 142 young stroke patients treated by the Department of Neurosurgery of Tianjin first Central Hospital from May 2019 to May 2021 were selected. Inclusion criteria: 1) In accordance with the diagnostic criteria of stroke established by the Fourth Academic Conference on Cerebrovascular Diseases, and further confirmed by imaging examination. 2) All patients were in a stable stage, aged from 18 to 45 years old, conscious and able to communicate normally. 3) They can cooperate to complete the treatment and assessment; 4) They are permanent residents of Tianjin (reached more than one year). Exclusion criteria: 1) Patients with serious diseases of other system organs in combination; 2) Patients with serious psychological disorders or psychiatric diseases. All patients were divided into a control group and an experimental group of 71 cases each according to the admission time order, and there was no statistically significant difference in general information between the two groups of patients and their caregivers (P > 0.05). The general information could be found in Tables 1 and 2.

**Table 1:** Comparison of general data between two groups of patients

Item		Experimental group (n=71)	Control group (n=71)	Statistics	*P* value
Age (years, x̅±s)		38.9±4.59	38.4±5.00	0.506^*^	0.355
Degree of education	Senior high school and below	26	24	0.770**	0.680
	Junior college or bachelor degree	40	44		
	Postgraduate and above	5	3		
Gender	male	48	44	0.494**	0.482
	female	23	27		
Disease type	Cerebral infarction	56	59	0.412**	0.521
	Cerebral hemorrhage	15	12		
Complication	High blood pressure	32	28	3.923**	0.270
	Diabetes	14	11		
	Hyperlipidemia	25	29		
Time from onset to hospital admission		2∼50h	1∼48h	0.227^*^	0.859
		(10.9±14.18)	(11.4±13.89)		

**Table 2:** Comparison of general data between the two groups of caregivers

Item		Experimental group (n=71)	Control group (n=71)	Statistics	*P* value
Age (years, x̅±s)		38.90±4.59	38.49 ± 5.00	0.862*	0.506
Degree of education	Senior high school and below	43	39	0.95**	0.622
	College or undergraduate	20	26		
	Graduate student or above	8	6		
Gender	male	27	23	0.494**	0.482
	female	44	48		
Relationship with patients	Partner	59	64	2.290***	0.130
	Parents	12	7		

* *t* value

** *χ^2^* value

*** Fisher exact probability method

## Procedure methods

### Establish a health education group and formulate a health education program

The health education team includes a department head, a head nurse, eight primary nurses, and four attending doctors. The department head was responsible for the development, supervision, and guidance of stroke-related work. Four attending physicians were responsible for the patients' surgery and treatment plan, and the head nurse was responsible for the whole stroke intervention and the quality control of the plan. Specific: 1 month to 1 year after discharge through monthly telephone follow-up and every six months group health lectures to understand the health behavior status of patients and conduct intervention. Five nurses were responsible for the propaganda and telephone return visit of cross-theoretical model health education, the other two nurses were responsible for the distribution and collection of questionnaires, and a residual nurse was responsible for health education diagnosis. In addition to the assistant of the health education group, the health education program for young stroke patients and caregivers in this study was developed elaborately in accordance with the researcher's own search of relevant literature at home and abroad.

## Nursing intervention method

### Nursing method of control group

In this group, the routine method of nursing in neurosurgical ward was adopted, and the changes of vital signs of patients were closely observed. The corresponding health education was carried out according to the disease process of each patient. The health education manual was issued upon discharge. This manual covers the characteristics of stroke diseases, how to do a good job of self-identification of stroke symptoms, the importance of blood pressure control, the importance of low salt diet for hypertensive patients, how to reasonably arrange diet and exercise for stroke patients, and the harmful of smoking and drinking alcohol on cerebrovascular disease, etc.

### Nursing method of experimental group

In this group, the intervention method of health education based on the cross-theoretical model was adopted. Through one-to-one questionnaire survey on the patients, their specific behavioral stage changes were evaluated. Accordingly, the corresponding health education diagnoses were given to the patients. On the basis of the above analysis, the researchers designed the specific health education intervention programs. Lastly, the missionary nurse implemented the appropriate health education program for the patients when they were in stable condition (Table S1).

**Table S1:** Health education intervention program based on the cross-theoretical model

hange stage	Change process	Empowerment objectives	Specific content of health education intervention	Intervention frequency
Pre-intentional stage	Consciousness Awakening	Build awareness of disease care	Patients and their families are introduced to what stroke is, the adverse effects of stroke on themselves, the effects of reoccurrence of the disease and common high-risk factors, pointing out the patients' currently adverse health problems and disease-related high-risk factors. Thus, patients can understand the symptoms of disease occurrence and seek timely medical attention.	The duration of each mission was 30 min per person 3 times per week.
	Vivid explanation		With the help of expert lectures, consultations and health talks, missionary nurses establish a good nurse-patient relationship with patients, gain their trust, educate patients and their families about stroke, inform them of the benefits of healthy behaviors for stroke, and provide them with authoritative medical information.	
Intention stage	Consciousness Awakening	Clarifying care issues and conducting family empowerment	Patients and family members were explained the role of establishing healthy behaviors to prevent the recurrence of stroke, especially the harmful effects of smoking on the cerebral vasculature. Health information related to stroke prevention was promoted through the WeChat public platform, and patients were encouraged to set health goals for themselves.	The duration of each mission was 20 min per person 2 times per week.
	Self-reevaluation		Evaluate the current lifestyle of patients, such as diet and exercise, encourage patients to evaluate their own lifestyle, and guide patients to actively talk about their bad lifestyle and how to improve it.	
	Environmental reevaluation		Understand the feelings of patients and their families after they get sick, focus on the harm caused by the patient's own bad health behaviors to the cerebrovascular. Explain the serious consequences of recurrent stroke, and make them realize that recurrence of stroke will significantly increase the burden on the family. Use successful cases to strengthen patients' determination in order to change their bad behaviors.	
Preparation stage	Self-liberation	Develop and establish a stroke health behavior plan	With the participation of patients and their families, we jointly develop a health behavior formation plan, including the expected goals, daily personal blood pressure monitoring, taking medicine on time,and the completion of review time, etc., in order to ensure the high quality completion of the health behavior change plan.	The duration of each mission was 20 min per person, 2 times per week.
	Social liberation		With the help of online lectures on stroke prevention knowledge held by the department, patients' self-management ability can be improved, and family members are encouraged to participate in stroke-related knowledge activities to increase patients' sense of social support.	
Action stage	Helping relationship	Implementation of stroke health behavior program	The missionary nurse establishes a good nurse-patient relationship with patients and their families during their hospitalization, asks about doubts in the process of changing undesirable behaviors, and gives timely guidance and encouragement. Establish patient groups, push stroke related knowledge web class lectures and improve related records, encourage patients to record their own diet exercise blood pressure and other conditions.	Once a month
	Anti-conditional effect		Based on the patient's daily records, we discuss with the patient about the current problems of the patient and give timely guidance to make adjustments properly.	
Retentive stage	Strengthen management	Effect evaluation and optimize nursing plan	Adhere to the importance of healthy behaviors to prevent stroke recurrence, make interview records, affirm and encourage patients to make correct behavioral changes, and encourage patients to continue to exert their subjective initiative and maintain good health behaviors	

During the intervention, if the patient's action stage was assessed to have regressed, then the patient's determination to change behavior will be reinforced by interviewing the patient to understand their reasons for the regression and also modeling and educating the patient by another patient with good behavioral change. This program was done in two sessions, including peer model motivational education and intra-group discussion (approximately 3060 min), with each person participating at least once.

## Evaluation tool

### Stroke Health Behavior Scale (HBS-SP)

The adopted scale was developed by Wan Lihong^10^ et al. and this scale is based on the Health Promotion Lifestyle Scale (HPLPII) proposed by Walker^11^, an American nurse practitioner. The used stroke health behavior scale (HBS-SP) is designed to assess the health behaviors of stroke patients in a disease-specific population. The content of this scale was divided into 6 dimensions with 25 items, and each question was scored on a 4-point Likert scale. 1=never, 2=sometimes, 3=often, 4=regularly, with a total score of 0-100. Noteworthy, the questions on the dimensions of tobacco, alcohol, and medication were scored inversely, with higher scores indicating better health behavior. In addition, the Cronbach's a coefficient of the scale was 0.878, and the content validity was 0.850, which has good reliability and validity.

### Behavior stage assessment questionnaire

The questionnaire used in this study was obtained from the American Center for Cancer Prevention^12^. The questionnaire, with a Cronbach's a coefficient of 0.76, asks patients to select an appropriate answer by asking whether they have established health behaviors as required, including the pre-intentional phase, by asking whether the patient was required to establish healthy behaviors (No, nor would it be considered for the next 3 months): Intention stage (no, but consider starting within 3 months): Preparation stage (No, but plan to start within the next 1 month and have adopted health protocol recommendations): Action stage (Yes, but the duration has not reached 3 months): Maintenance stage (Yes, it has been for more than 3 months).

### Positive Aspects of Caregiving Scale (PAC scale)

This scale was proposed by Tarlow^13^ to evaluate the positive feelings of caregivers with good reliability and validity. It consists of self-affirmation (5 items) and life outlook (4 items), with 2 dimensions, on a 5-point Likert scale, with scores from 1 to 5 representing “strongly disagree to strongly agree”, and a total score of 9 to 45, with higher scores indicating higher caregiver feelings.

### Data collection method

A questionnaire survey was conducted within 3 days before admission, 1 month, 3 months, and 6 months after intervention. The purpose and content of the questionnaire were explained in detail for the patients before the survey, and also the informed consent form was signed. When patients have doubts about the contents of the questionnaire, consistent language is used to explain. The questionnaire was collected by the combination of on-site and online questionnaire, and the main caregiver questionnaire was sent online for evaluation and collection after intervention within 3 months.

### Statistical methods

In this work, the SPSS23.0 statistical software was used for the statistical analysis. Quantitative data were described by x̅±s, and the health behavior scores were described by two-sample t test. The *χ***^2^** test was used for categorical data and the comparison of behavioral stage changes. Furthermore, the analysis of variance was used for the comparison of blood pressure values between the two groups, with P<0.05 being considered statistically significant difference.

## Result

### Comparison of health behaviors between the two groups before and after intervention

Before the intervention, there was no statistically significant difference between the health behaviors of the two groups (P>0.05). However, after 1 month of the intervention, there was a statistically significant comparison between the dimensions of nutrition and blood pressure monitoring in the health behaviors of the two groups (P<0.05). Moreover, after 3 and 6 months of the intervention, there was a statistically significant comparison between the scores of each dimension of the health behaviors of the two groups (P<0.05), as shown in Table 3.

**Table 3:** Comparison of stroke health behavior scale scores between two groups before and after intervention. (Points, x̅±s)

Time	Group	Exercise	Nutrition	Medication compliance	Low-salt diet	Blood pressure monitoring	Quit smoking and limit alcohol
Before intervention	Experimental group	10.83±3.62	9.27±3.02	9.34±2.95	1.73±0.97	1.49±0.80	5.37±2.61
	Control group	10.94±3.63	9.52±2.95	9.61±3.04	1.75±0.85	1.66±0.81	5.61±2.42
	*t*	0.185	-0.506	-0.531	-0.092	-1.245	-5.66
	*P*	0.854	0.614	0.596	0.927	0.215	0.572
Intervention for 1 month	Experimental group	11.93±3.87	16.45±4.32	10.15±3.15	1.86±0.93	2.06±0.82	5.72±2.46
	Control group	10.82±3.51	14.83±4.19	9.68±3.01	1.79±0.84	1.75±0.77	6.11±2.11
	*t*	1.560	2.26	0.925	0.472	2.313	-1.024
	*P*	0.120	0.025*	0.350	0.637	0.022*	0.308
Intervention for 3 months	Experimental group	13.90±4.29	18.35±5.69	11.54±3.08	2.46±1.23	2.37±0.91	6.76±1.74
	Control group	11.28±3.64	15.90±5.19	10.04±2.94	1.89±0.87	1.86±0.72	6.07±2.06
	*t*	3.921	2.678	2.948	3.231	3.666	2.151
	*P*	0.000*	0.008*	0.004*	0.002*	0.000*	0.033*
Intervention for 6 months	Experimental group	17.62±6.03	22.58±7.18	11.75±3.09	2.96±0.95	3.31±0.77	7.32±1.21
	Control group	11.55±3.86	16.15±4.96	10.49±2.65	2.06±0.97	1.76±0.75	5.75±2.31
	*t*	7.138	6.199	2.596	5.603	12.203	5.092
	*P*	0.000*	0.000*	0.010*	0.000*	0.000*	0.000*

Note

* *P*<0.05.

### Comparison of behavioral stage changes between the two groups before and after intervention

As shown in Table 4, compared with the control group, the experimental group had more patients in the action stage and maintenance stage. There was significant difference in the behavioral stage changes of patients between the two groups at different time points (P<0.01).

**Table 4:** Comparison of the stages of behavior change of patients at different time points of the intervention between the two groups [Case]

Group	Time	Case	Pre-intentional stage	Intentional stage	Preparation stage
Control group	Before intervention	71	47	14	6
	One month after intervention		20	18	16
	3 months after intervention		18	21	18
	6 months after intervention		11	30	24
Experimental group	Before intervention	71	44	17	5
	One month after intervention		19	14	16
	3 months after intervention		10	17	16
	6 months after intervention		2	8	10

Note: Comparison of two groups at different time points, *Z*=-1.014, -0.169, -0.768, 1.951; *P*=0.000, 0.000, 0.000, 0.000

### Comparison of positive feelings of main caregivers between the two groups before and after intervention

The detail comparison results of positive feelings of main caregivers between two groups before and after intervention was shown in Table 5. Results showed that the positive feeling scores of primary caregivers in the experimental group were significantly higher than those in the control group after the intervention (P<0.01).

**Table 5:** Comparison of positive feelings of main caregivers between two groups before and after intervention

Group	case	Self-affirmation				Life outlook			

		Before intervention	After intervention	*t*	*P*	Before intervention	After intervention	*t*	*P*
Control group	71	8.79 ± 3.33	8.96 ± 3.19	-4.06	0.686	8.55± 3.29	8.76 ± 3.34	-1.534	0.129
Experimental group	71	8.63 ± 3.15	15.14±3.20	-19.615	0.000	8.11±2.73	14.99±3.34	-21.522	0.000
*t*		0.569	-9.227			0.858	11.148		
*P*		0.57	0.000			0.392	0.000		

## Discussion

### Family empowerment health education based on a cross-theoretical model facilitates the establishment of health behaviors in young stroke patients

In this study, the scores of each dimension of health behavior in the experimental group at 3 months and 6 months after the intervention were higher than those in the control group, and the difference was statistically significant (P<0.05). It is beneficial for young stroke patients to establish healthy behaviors, and at the same time, long-term guidance and supervision are necessary to help patients develop healthy behaviors. In general, the recurrent nature of stroke seriously affects the ability of young stroke patients to return to work, family, and social life^14^. Studies have shown that a healthy lifestyle with appropriate physical activity, a healthy diet, low-salt foods, and abstinence from smoking and alcohol can prevent the occurrence and recurrence of stroke^15^-^18^. In addition, knowledge lectures on specific topics, for instance, risk factors that endanger young stroke patients, how to protect vascular health, and the harm of smoking and drinking to the body, coupled with peer motivation, family member support and medical care, are indeed effective to help patients establish healthy behaviors properly. Therefore, we can conclude that health education based on a cross-theoretical model can play an important role in establishing healthy behaviors in young stroke patients by targeting interventions to patients' own problems through the behavioral stage they are in, and giving individualized guidance. The 2022 US National Standards for Diabetes Self-Management Education^19^ clearly indicate that more support and follow-up is needed to maintain effective self-management behaviors. In addition, the introduction of the concept of self-efficacy in this model can increase patients' confidence in a particular situation. According to the self-efficacy theory, the failure experience of others will reduce the self-efficacy of individuals, while the successful experience can improve the self-efficacy of individuals^20^. Therefore, in the intervention process, patients in the maintenance stage are invited to explain the successful experience after adhering to a healthy lifestyle and bringing the disease under control, so as to improve the patients' self-efficacy and self-protection confidence.

### Implementation of a cross-theoretical model-based family empowerment health education effectively promotes behavioral stage changes in young stroke patients

The results of this study showed that the behavioral stage changes after the intervention of the young stroke patients in the experimental group were better than those in the control group (P<0.01). The experimental group had more patients in the action stage and maintenance stage. By dividing the patients with different behavioral stages, targeted programs were adopted to guide patients to change their behavior. In the pre-intentional stage and intentional stage, we help patients understand that stroke is a disease that is inseparable from lifestyle through explanations, examples, expert presentations, and media communication. Thus, changing one's poor lifestyle plays an important preventive and control role in preventing stroke recurrence. In the preparation stage, the synergy of the patient's family members should be fully mobilized to jointly formulate a detailed behavior change plan, and the feasibility of behavior change of the patient would be enhanced by issuing reminder kits, blood pressure self-monitoring logs, etc. Results showed that the number of people in the experimental group increased significantly in the action and maintenance stages, and the difference was statistically significant (P<0.01), which proved that most of the young stroke patients in the experimental group could consciously establish healthy behaviors after intervention. Foreign research by Sullivan et al.^21^ found that the health beliefs of stroke patients played an important role in changing unhealthy behaviors to reduce the risk of stroke recurrence. Therefore, the removal of possible risk factors can play a promoting role in preventing or delaying the occurrence of cerebrovascular events, reducing the number of re-hospitalizations, reducing economic burden, and improving the quality of life.

### Family empowerment health education based on a cross-theoretical model can promote the degree of positive feelings of caregivers

The uncertainty of the caregiver will seriously affect the caregiver's outlook on life and also the outcome of the patient's care^22^. Obviously, the positive feelings of the caregiver are of great significance to improve the quality of life of the corresponding patient^23^. Generally, stroke patients need long-term family support to build a health management model^24^. This study used a cross-theoretical model of family empowerment health education to maximize family initiative, improve the motivation level, and psychological state of caregivers, promote the effective implementation of health behavior promotion program for stroke patients, and ultimately facilitate the formation of health behavior. The results of this study showed that the positive feeling scores of primary caregivers in the experimental group were significantly higher than those in the control group after the intervention (P<0.01), indicating that the family empowerment education research through the cross-theoretical model was effective in motivating caregivers to take the initiative to learn about stroke and improve their knowledge about stroke, thus improving their ability to cope with the disease. By discussing with caregivers and guiding caregivers to actively participate in patient health management, caregivers increase their sense of self-affirmation and give full play to their self-efficacy. At the same time, caregiver self-efficacy enhancement contributes to patient health behavior supervision and also enhances intimacy with patients, which gives caregivers new expectations for their lives. In short, the cross-theoretical model is a purposeful behavior change model. The model emphasizes that individual behavior change is a continuous process. Before real behavior change, people develop towards a series of dynamic and cyclic changes. For individuals at different stages, different strategies should be adopted to promote their transition to action and maintenance stage^1^. Studies have shown that interventions based on cross-theoretical models have a significant effect on improving patients' bad behavior^25^,^26^.

## Conclusion

Family empowerment health education based on a cross-theoretical model promotes the establishment of health behaviors and the process of behavior stage change in young stroke patients, which also helps to increase the degree of positive feelings of primary caregivers and maximizes the patient's ability to self-manage the disease. Thus, the present cross-theoretical model-based family empowerment health education in this study is an effective health education method. However, this study did not provide the long-term contact and communication with patients due to limited conditions. We will continue to explore the long-term effective forms of intervention to provide timely and effective guidance to patients in anticipation of better clinical outcomes.

## References

[1] Katan M, Luft A (2018). Global Burden of Stroke. Semin Neurol.

[2] Saei Ghare Naz M, Simbar M, Rashidi Fakari F, Ghasemi V (2018). Effects of Model-Based Interventions on Breast Cancer Screening Behavior of Women: A Systematic Review. Asian Pac J Cancer Prev.

[3] Zare M, Tarighat-Esfanjani A, Rafraf M, Shaghaghi A, Asghari-Jafarabadi M, Shamshiri M (2020). The Barriers and Facilitators of Self-Management Among Adults with Type 2 Diabetes Mellitus: A Trans Theoretical Model (TTM)-Based Mixed Method Study in Iran. Diabetes Metab Syndr Obes.

[4] Lu Y, Yi S (2007). Logistic regression analysis of cerebral infarction recurrence factors[J]. Natural Science Journal of Hunan Normal University.

[5] Chen C (2000). Discussion on risk factors of stroke recurrence and prevention [J]. Journal of Guangxi Medical University.

[6] Abdi J, Eftekhar H, Mahmoodi M, Shojaeizade D, Sadeghi R (2015). Lifestyle of the employees working in hamadan public sectors: application of the trans-theoretical model. Iran Red Crescent Med J.

[7] Liu YS, Ren HY (2019). [The application progress of trans-theoretical model on changing the healthy behavior of patients with coronary heart disease]. Zhonghua Xin Xue Guan Bing Za Zhi.

[8] Lutz W, Schwartz B (2021). Trans-theoretical clinical models and the implementation of precision mental health care. World Psychiatry.

[9] Yin B (2007). A cross-theoretical model of health behavior change [J]. Chinese Journal of Mental Health.

[10] Wan Lihong, Xiong Xiaoni, Pan Junhao (2017). Development, reliability and validity of health behavior scale for patients with stroke [J]. Journal of Nursing.

[11] Walker SN, Sechrist KR, Pender NL (1987). The health promoting lifestyle profile development and psychometric characteristics[J]. Nurs Res.

[12] Luann R (2012). Motivational interviewing: helping patients move toward change [J]. J Christ Nurs.

[13] Tarlow BJ (2004). Positive aspects of caregiving: contributions of the REACH Project to the development of new measures for Alzheimer' caregiving[J]. Res Age Intern Bim J.

[14] Guo Yawen, Wang Yongli, Liang Lili (2020). Research status and enlightenment of young and middle-aged people returning to work after stroke [J]. Nursing Research.

[15] Cheng Xiaoqian (2018). To explore the effect of health risk behaviors on stroke prevention in young people [J]. Chinese Journal of practical Neurological Diseases.

[16] O'donnell MJ, Chin SL, Rangarajan S (2016). Global and regional effects of potentially modifiable risk factors associated with acute stroke in 32 countries (INTERSTROKE): a case-control study[J]. Lancet.

[17] Chen Pang (2020). Study on the relationship between positive psychological capital, coping style and health behavior in young and middle-aged patients with stroke.

[18] Chen Juan, Dong Mingxia, Yang Qi (2019). Analysis of health behavior and its influencing factors in young stroke patients [J]. Chinese Health Statistics.

[19] Davis J, Fischl AH, Beck J, Browning L, Carter A, Condon JE, Dennison M, Francis T, Hughes PJ, Jaime S, Lau KHK, McArthur T, McAvoy K, Magee M, Newby O, Ponder SW, Quraishi U, Rawlings K, Socke J, Stancil M, Uelmen S, Villalobos S (2022). 2022 National Standards for Diabetes Self-Management Education and Support. Sci Diabetes Self Manag Care.

[20] Jiang X, Xue Y, Wang G (2004). Research Progress in Self-efficacy [J]. Nursing Research.

[21] Sullivan KA, White KM, Young RM, Scott C, Mulgrew K (2008). Developing a stroke intervention program: what do people at risk of stroke want?. Patient Educ Couns.

[22] Lin S, Wang Q, Xie S (2022). The experience of stroke survivers and caregivers during hosptial to home trasitional care:A qualitative longitudinal study[J]. Int J Nurs Stud.

[23] Peyrovi H, Mosayebi Z, Mohammad-Doost F (2016). The effect of empowerment program on “perceived readiness for discharge” of mothers of premature infants[J]. J Matern Fetal Neonatal Med.

[24] Pindus D M, Mullis R, Lim L (2018). Stroke survivors' and informal caregivers' experiences of primary care and community healthcare services–a systematic review and meta-ethnography[J]. PloS One.

[25] Huang J, Wang L, Cao X (2012). Effect of cross-theoretical model on health education of elderly patients with hypertension [J]. Journal of Nursing Science.

[26] Erol S, Erdogan S (2008). Application of stage besed motivational interviewing approach to adolescent smoking cessation: the Transtheoretical Model-based study. Patient Educ Couns.

